# *QuickStats:* Number of Deaths Resulting from Being Bitten or Struck by a Dog,* by Sex — National Vital Statistics System, United States, 2011–2021

**DOI:** 10.15585/mmwr.mm7236a6

**Published:** 2023-09-08

**Figure:**
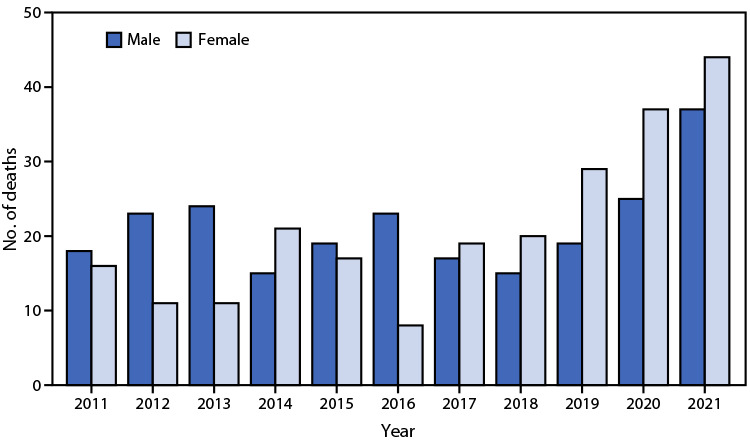
During 2011–2021, a total of 468 deaths from being bitten or struck by a dog occurred (average = 43 deaths per year). The annual number of deaths ranged from 31 (2016) to 81 (2021). During 2011–2016, more deaths occurred among males than among females during most years; however, during 2017–2021, more deaths occurred among females than among males. From 2018 to 2021, deaths more than doubled for both males (from 15 to 37) and females (from 20 to 44).

**Source:** National Center for Health Statistics, National Vital Statistics System, Mortality Data, 2011–2021. https://wonder.cdc.gov/Deaths-by-Underlying-Cause.html

